# Influence of Textural Properties of Co@SiO_2_ Catalysts on the Performance of Fischer–Tropsch Synthesis

**DOI:** 10.1021/acsomega.5c04178

**Published:** 2025-09-30

**Authors:** Abigail N. E. Ojeda, Camylla M. Moraes, Alexander E. Caytuero-Villegas, Maria Auxiliadora S. Baldanza, Fabio S. Toniolo, Neuman S. de Resende, Vera M. M. Salim

**Affiliations:** Chemical Engineering Program/COPPE, Centro de Tecnologia, 28125 Universidade Federal do Rio de Janeiro (UFRJ), Cidade Universitária, Rio de Janeiro 21941-901, Brazil

## Abstract

Cobalt core–shell catalysts are advanced materials for Fischer–Tropsch synthesis, which enhance catalytic performance. This study explores the impact of pore diameter on the performance of mesoporous silica-coated cobalt oxide core–shell catalysts (Co@SiO_2_), maintaining a constant shell thickness while varying the porosity. The synthesis method successfully yields a core–shell morphology with a centralized core, an average core diameter ranging from 30 to 34 nm, a total diameter between 97 and 103 nm, and a SiO_2_ shell thickness of 32.5–34.5 nm. Textural characterization shows a specific surface area from 236 to 771 m^2^/g, with average pore diameters between 3.5 and 11.8 nm. The results indicate a significant change in the distribution of products, exhibiting more expressive selectivity in the range of C_19_–C_24_ and C_25_–C_29_ hydrocarbons with the core–shell catalyst and C_30+_ hydrocarbons with the supported catalyst. It also demonstrates that a decrease in pore diameter of the silica shell leads to significant changes in product distribution. Specifically, a reduction in the pore size from 9.2 nm (Co@SiO_2__0.03) to 3.5 nm (Co@SiO_2__0.09) results in a 17% increase in selectivity toward the C_19_–C_24_ fraction and a concomitant 24% decrease in the C_25_–C_29_ fraction. Furthermore, the silica shell provided a protective effect on the active phase, with no evidence of sintering observed in the spent encapsulated catalysts.

## Graphical abstract

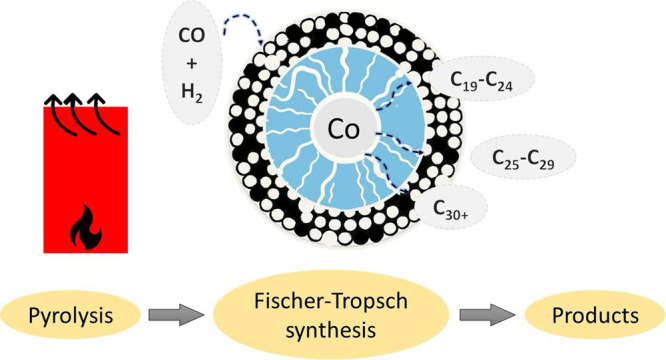

## Introduction

The urgent requirement to reduce greenhouse gas emissions is a critical challenge of the 21st century. Such actions are essential to avert imminent environmental collapse and safeguard the long-term survival of humanity and the planet.

In this scenario, Fischer–Tropsch synthesis (FTS) has gained renewed interest due to its potential to produce clean liquid fuels via the gas-to-liquid (GTL) process. A century after its discovery in Germany by Franz Fischer and Hans Tropsch, Fischer–Tropsch Synthesis (FTS) stands out as a remarkable process for producing clean liquid fuels via the gas-to-liquid (GTL) route. As is well-known, FTS is an established technology, with commercial-scale plants successfully operating to produce clean fuels with low sulfur and aromatic content, using alternative petroleum resources like natural gas.^1–3^ Currently, the challenges in improving FTS technology include enhancing carbon efficiency through reduced energy consumption, minimizing CO_2_ emissions, and achieving better selectivity control.^4,5^ From this perspective, biomethane and CO_2_ as raw materials have emerged as promising pathways for the sustainable production of fuels.^6,7^

Cobalt catalysts for FTS are well-known for their remarkable activity at low temperatures, enabling the production of long-chain paraffins, lower susceptibility to the water gas shift reaction (WGSR), and better resistance to deactivation by water. Other supports such as Al_2_O_3_ and TiO_2_ are widely employed and well studied in the literature; however, in this work, Co_3_O_4_ was chosen as the support.^8,9^ Aiming for the optimization of product yields, parameters such as the nature of the support, the textural properties of both the support and catalyst, the nature of the active phase, and reaction conditions are being revisited, with a deeper understanding being developed, particularly with a focus on process intensification.^10–13^ Focusing on the catalytic performance optimization of product yields requires activity improvement, selectivity control, and minimization of catalyst deactivation.^14–18^

The activity improvement requires a more precise identification of the nature of the active phase. This subject remains imprecise and continues to be an area of research, attracting academic and industrial interest.^12,19^ The metallic cobalt is widely accepted as the primary active phase. However, other phases, such as cobalt oxides, cobalt carbides, cobalt with carbon deposits, and cobalt-support interfaces, have also been considered.^9,11,20^ Moreover, the support nature and syngas composition feed strongly affect the active cobalt phase under FTS reaction conditions. For instance, in a conventional syngas feed with a H_2_/CO molar ratio of 2 and silica supports, the metallic cobalt is predominantly regarded as the main active phase.

Regarding the crystallographic reaction, the hexagonal close-packed (HCP) and face-centered cubic (FCC) are the primary crystallographic allotropes of the cobalt nanoparticle phase under the FTS reaction. Theoretical studies indicated that cobalt in the HCP phase exhibits higher stability against carbon deposition and better anticarbonization capability than Co in the FCC phase due to the higher density of active sites.^21^

Studies on the structure sensitivity of FTS have identified an optimal cobalt nanoparticle size in a range of 6 to 12 nm. Particles larger than this range show a lower turnover frequency number (TOF), while smaller particles produce more methane and are more susceptible to reoxidation. The support material affects the performance of the cobalt catalysts as it affects the dispersion, reducibility, and stability of the active metal center. SiO_2_ is a commonly used oxide support due to the relatively weak metal–support interaction (MSI), which promotes a higher degree of reduction of the cobalt species. However, this weak interaction requires careful adjustment to avoid large cobalt particle formation, which can reduce metal dispersion and compromise thermal stability.^19,22–25^

The textural properties of mesoporous support significantly affect the catalytic performance through changes in particle morphology, dispersion, reducibility, and mass transfer properties.^24,26–28^ For instance, increasing Co/SBA-15 pore size from 3 to 15 nm enhanced the catalytic activity, such as a larger pore diameter, improving C_5+_ selectivity. However, further increases in pore size have been found to exert a limited influence on the activity.^29^ Furthermore, the nature of the support also affects both the thermal conductivity and the reducibility of the precursor. Both are critical parameters that affect the catalytic performance. Lower reducibility, in particular, may contribute to reduced activity and improve CH_4_ selectivity.

Catalytic deactivation phenomena, including cobalt particle sintering and the formation of inactive compounds, such as cobalt carbide, oxide, and silicate through spontaneous reactions at the metal–support interface under reaction conditions, must be mitigated to extend the catalyst cycle life. Wolf et al. and Wolf et al. identified the Co_2_SiO_4_ phase in all spent catalysts via X-ray absorption spectroscopy, addressing these deactivation processes.^27,30^ Similarly, Zhou et al. observed silicate species in spent catalysts using TPR characterization.^31^ Furthermore, Puskas et al. noted that the spinel structure of Co_2_SiO_4_ closely resembles that of Co_3_O_4_, indicating the potential coexistence of spinel and silicate phases in the spent catalysts.^32^

Core–shell morphology is emerging as a promising next-generation catalyst design for Fischer–Tropsch synthesis (FTS). It offers the ability to mitigate sintering processes while effectively modifying product distribution compared with conventional supported catalysts. This is achieved through precise adjustments to the metal size and the shell textural properties.^22,33–37^

Cobalt nanoparticles can be synthesized by using a solvothermal method and subsequently encapsulated in silica via a modified Stöber method. This approach yields catalysts with a well-centralized core and a silica shell whose textural properties can be tailored.^33^ Notably, the silica shell thickness plays a critical role in the reaction selectivity. Zeng et al. investigated the effect of shell thickness (ranging from 4 to 18 nm) on C_5_–C_18_ selectivity on 14.7% Co@SiO_2_ catalysts, identifying 12.5 nm as the optimal thickness.

Currently, the optimization of catalyst geometry to overcome the selectivity limitations of Fischer–Tropsch synthesis is an area of extensive research. The use of hexadecyltrimethylammonium bromide (CTAB) as a templating agent and the impact of varying hydrothermal and pyrolysis temperatures on the morphology and structure of the Co@SiO_2_ core–shell were systematically investigated. These adjustments were correlated with changes in catalytic activity and product selectivity.^36,39^

In our previous work, we explored the preparation of Co@SiO_2_ catalysts with varying shell thicknesses under different reaction conditions, aiming to optimize selectivity for the desired fraction.^34^ Notably, the shell thickness revealed a significant impact on the reaction selectivity. Catalysts with thinner shells exhibited higher selectivity toward C_19_–C_24_ hydrocarbons, whereas those with thicker shells showed increased selectivity toward methane. This behavior is likely attributed to the limitations of CO diffusion in catalysts with thicker shells.

In this work, we investigate the influence of the pore diameter of the silica shell in Co@SiO_2_ catalysts on their performance in Fischer–Tropsch synthesis while maintaining a constant shell thickness and metal particle size. Hexadecyltrimethylammonium bromide (CTAB) was employed as a templating agent to adjust pore-size diameter. All synthesized samples exhibited well-dispersed particles with uniform size, cores with a mean diameter of approximately 30 nm, and a total mean diameter of around 100 nm.

## Experimental Section

### Catalyst Preparation

#### Preparation of the Supported Catalyst, Co/SiO_2_

The reference catalyst was prepared using the incipient wetness impregnation using a Co­(NO_3_)_2_.6H_2_O (98%) aqueous solution. First, 3.04 g of fully dissolved cobalt nitrate in 5 mL of water (to get 6% mass content) was slowly added to 9.18 g of SiO_2_ until the dry impregnation point was reached in a single step. The obtained material was dried at 120 °C for 24 h, and then it was calcined at 400 °C for 3 h at 1 °C/min of heating rate.

#### Preparation of a Core–Shell Catalyst, Co@SiO_2_

To obtain Co3O4 nanoparticles, the core–shell nanostructured catalyst was prepared by the solvothermal process and the modified Stöber process for its encapsulation by a silica shell. First, 1.77 g of cobalt­(II) nitrate hexahydrate (Co­(NO_3_)­2.6H_2_O, 98%) and 3.55 g of polyvinylpyrrolidone (PVP) were dissolved in 200 mL of ethanol under magnetic stirring. The solution was transferred into a Teflon-lined stainless-steel autoclave, which underwent thermal treatment in an oven at 180 °C for 4 h. Then, after it naturally cooled to room temperature, the obtained suspension was transferred into a round-bottom flask, and 519 mL of ethanol, 414 mL of distilled water, and 32 mL of ammonium hydroxide were added to the suspension.

The influence of the structure-directing agent cetyltrimethylammonium bromide (CTAB) on the textural properties of the silica layer was evaluated by varying the amounts used (0.7518, 1.50, 1.87, and 2.25 g). The mixture was vigorously stirred for 2 h at room temperature. Then, 15.3 mL of tetraethylorthosilicate (TEOS) was dripped into the mixture at a flow rate of 0.01 mL/min by using a syringe pump. After the addition of TEOS, the mixture remained under vigorous stirring for 48 h. Throughout the synthesis, the pH remained at 11, confirming that the medium was strongly basic. The material was separated by centrifugation at 10,000 rpm for 1 h and washed with distilled water and ethanol. The obtained solid was dried at 80 °C for 24 h, and then it was calcined under an air flow at 500 °C with a heating rate of 10 °C min^–1^ for 6 h. The samples obtained were coded as Co@SiO_2__*x*, where *x* represents the CTAB/TEOS molar ratio, as indicated in Table 1.

**Table 1:** CTAB Mass, CTAB/TEOS Molar Ratio, and Coding of Samples to Each Catalyst

CTAB weighed mass (g)	CTAB/TEOS molar ratio	coding of samples after calcination
0.75	0.03	Co@SiO_2__0.03
1.55	0.06	Co@SiO_2__0.06
1.87	0.075	Co@SiO_2__0.075
2.25	0.09	Co@SiO_2__0.09

### Catalyst Characterization

#### N_2_ Physisorption

Textural properties, the specific surface area (Sg), the average pore diameter (*d*_p_), and the pore volume (*V*_p_) of the calcined catalysts were determined via N_2_ adsorption at 77 K using the ASAP (Accelerated Surface Area and Porosity) equipment, model 2010 from Micromeritics, after pretreating the samples under vacuum at 200 °C for 14 h. The Brunauer, Emmett, and Teller (BET) adsorption method was used to determine the surface area (*S*_g_), and the Brunauer, Joyner, and Halenda (BJH) method was used for the determination of pore volume (*V*_p_) and average pore size (*d*_p_).

#### X-ray Diffraction

X-ray diffraction (XRD) analyses of the calcined catalysts were performed with a Rigaku Miniflex X-ray diffractometer using monochromatic Cu–K_α_ radiation (λ = 1.5418 Å). The operating conditions for analysis were: 2θ angular sweep from 10 to 80°, angular step of 0.05°, and step time equal to 2 s. The Co_3_O_4_ average crystallite size (*d*_Co3O4_) was estimated by the Scherrer equation. The Co^0^ crystallite size (*d*_dCo0_) was calculated according to eq 1 ^40^ considering the relation between the relative molar volumes of Co^0^ and Co_3_O_4_:

$$d_{{\mathrm{Co}}^{0},\mathrm{XRD}}=0.75d_{{\mathrm{Co}}_{3}O_{4},\mathrm{XRD}}$$ 1

Cobalt dispersion (*D*) was assessed by correlating it with the Co^0^ crystallite size determined through XRD (eq 2),^40^ assuming spherical particle geometry and a site density of 14.6 atoms nm^–2^.

$$D(\%)=\frac{96.2}{d_{{\mathrm{Co}}^{0},\mathrm{XRD}}(\mathrm{nm})}$$ 2

#### Scanning Transmission Electron Microscopy

The morphology was investigated by a field emission gun scanning electron microscope (FEG-SEM), a FEI Tecnai Nanolab G20 FEG. The scanning transition electron mode on an SEM microscope (STEM-in-SEM) under SEM low voltage (30 kV) operating conditions increased resolution. The configuration for STEM-in-SEM includes a high-angularity STEM detector (HAADF-STEM) below the sample holder. Samples were suspended in ethanol and dispersed ultrasonically for 60 min. A drop of the suspension was deposited on a copper grid coated with a holey carbon film. Particle size measurements were performed using Image-J (1.52a). Over 400 particles were taken for statistical purposes to determine the total average diameter and core of the encapsulated nanoparticles.

#### Temperature-Programmed Reduction

Temperature-programmed reduction (TPR) was carried out with approximately 100 mg of the catalyst in a quartz reactor, under a flow of 50 mL/min of pure H_2_ at a heating rate of 5 °C/min up to a temperature of 1000 °C. The experiment was monitored using a Pfeiffer Vacuum mass spectrometer, model QME 200, monitoring the signal relative to the water ion, *m*/*z* = 18. The samples were pretreated with a stream of pure helium (30 mL/min) at 200 °C for 1 h. After this step, the catalyst was cooled to room temperature.

### Catalytic Activity

Fischer–Tropsch synthesis was carried out in a fixed-bed vertical tubular reactor loaded with 300 mg of the catalyst. The catalysts were reduced in situ at 400 °C with a heating rate of 5 °C min^–1^ for 10 h under a flow of 50 mL min^–1^ of pure H_2_. Afterward, the system was cooled to the feeding temperature under a hydrogen gas atmosphere, and then a flow of 50 mL min^–1^ of He passed through the catalytic bed for 30 min. Next, the feed gas was added, composed of a H_2_/CO/N_2_ gas mixture (60:30:10 v/v), in which N_2_ was the internal standard. The reaction effluent was analyzed by an online gas chromatograph (Shimadzu 2010), with a flame ionization detector (FID) and a barrier ionization discharge detector (BID).

The performance tests with core–shell and supported catalysts were carried out under isothermal conditions (210 °C) and pressure of 2 MPa, with a gas hourly space velocity (GHSV) of 10,000 mL g_cat_^–1^ h^–1^, H_2_/CO molar ratio of 2, and time-on-stream of 72 h.

The CO conversion (*X*_CO_) and the selectivity (*S*_*i*_) were calculated by the following equations ( eqs 4 and 5):

$$m(\%)=100\times \frac{A_{i}}{\sum A_{i}}$$ 3

$$X_{\mathrm{CO}}=100\times \frac{\left(1-\frac{A_{\mathrm{CO}}}{A_{N_{2}}}\right)}{\left(\frac{\mathrm{CO}}{N_{2}}\right)}$$ 4

$$S_{i}(\%)=100\times \frac{\left(\frac{m_{i}}{M_{i}}\right)}{\sum \left(\frac{m_{i}}{M_{i}}\right)}$$ 5

where *i* is the carbon number, *A_i_* is the area corrected with carbon number *i*, *m* is the mass percentage, and *M_i_* is the molar mass of the alkane with carbon number *i*.

## Results and Discussion

### Catalyst Characterization

The textural properties of the calcined core–shell catalysts, along with the Co/SiO_2‑_supported catalyst used as a reference, are summarized in Table 2. The N_2_ adsorption–desorption isotherms of both the supported and encapsulated catalysts correspond to type IV, characteristic of mesoporous materials, with hysteresis loops indicative of capillary condensation within the mesopores. All samples exhibit an average pore size within the mesoporous range (2 to 50 nm), consistent with the observed isotherm patterns. The results reveal the specific area covering a range of 236–771 m^2^/g and an average pore diameter of 3.5–11.8 nm, values deemed suitable for assessing the influence of pores on catalytic selectivity. The Supporting Information provides the adsorption isotherms of the supported and encapsulated catalysts.

**Table 2:** Textural Properties of the SiO_2_ Support and Calcined Catalysts

catalyst	Co content (wt %)	*S*_g_ (m^2^/g)	*V*_p_ (cm^3^/g)	*d*_p_ (nm)
SiO_2_		268	1.1	10.9
Co/SiO_2_	4.9	236	0.95	11.8
Co@SiO_2__0.03	7.3	300	0.56	9.2
Co@SiO_2__0.06	6.3	640	1.02	5.9
Co@SiO_2__0.075	7.3	739	1.22	5.9
Co@SiO_2__0.09	7.1	771	0.83	3.5

These results show that the increase of CTAB concentration (from sample Co@SiO_2__0.03 to Co@SiO_2__0.09) raises the specific area while reducing the pore diameter, yielding mesoporous materials with diverse pore sizes. The findings are consistent with previous reports, which demonstrate that a strong interaction between PVP and the surfactant cations (CTA+) is highly effective in forming the mesoporous silica shell.^36,41^ The key challenge addressed in this work is the synthesis of catalysts with varying pore diameters while maintaining the same core diameter for the active phase and the total mean diameter. These textural properties obtained are essential to the goals of this study.

Scanning transmission electron microscopy (STEM) analysis confirmed the successful synthesis of our catalysts via a solvothermal method. As depicted in Figure 1, all the encapsulated catalysts exhibit a well-defined core–shell morphology with a centralized metallic nucleus. The core–shell structure is observed, as well as the active phase nucleus composed of aggregates of crystallites. These aggregates contribute to an increased metallic surface area, which may enhance the catalytic performance. Moreover, the Co@SiO_2_ particles are uniformly dispersed, showing no significant aggregation.

**Figure 1:**
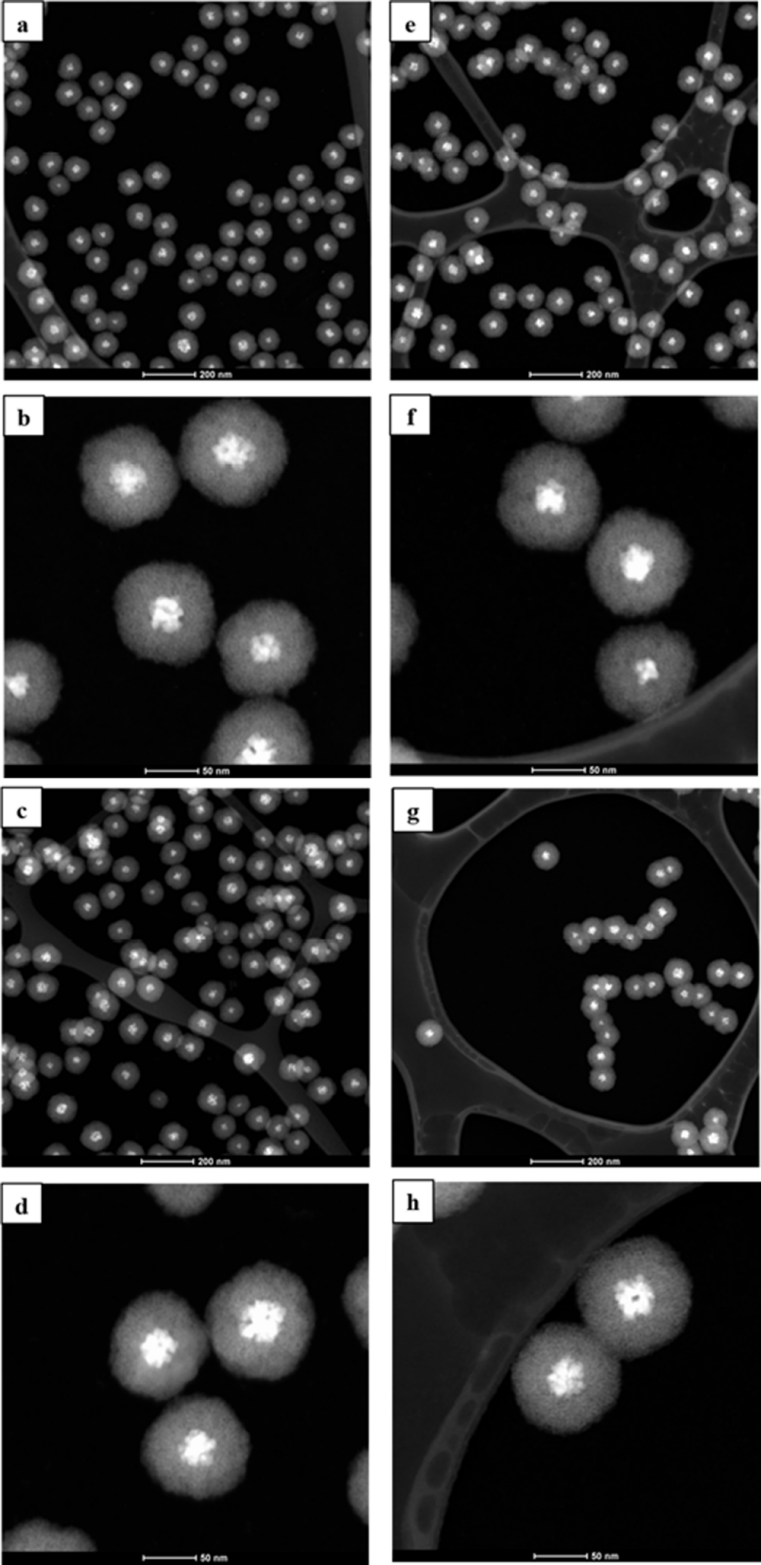
STEM images of the Co@ catalysts samples: Co@SiO_2__0.03 (a, b), Co@SiO_2__0.06 (c, d), Co@SiO_2__0.075 (e, f), and Co@SiO_2__0.09 (g, h).

According to Table 3, all samples exhibit a uniform size, with an average total diameter in the range of 100 nm and a core of approximately 30 nm. Additionally, the average thickness of the silica shell was around 33 nm. It can be observed that varying the surfactant concentration during the encapsulation step did not change the size or the morphology of the cobalt oxide core, as previously observed in the literature.^42^ Moreover, the increase in the CTAB concentration does not lead to significant variation in the thickness of the silica shell. Reports of the literature suggest that increasing the concentration of CTAB results in the formation of a greater number of micelle structures, which decreases the amount of TEOS precursor available per surfactant molecule, leading to the formation of smaller particles.^42,43^ However, in the range of concentrations used here, changes in silica thickness are not noticeable.

**Table 3:** Average Diameter of Core–Shell Nanoparticles after Calcination

samples	core diameter (nm)	total diameter (nm)	SiO_2_ shell thickness (nm)
Co@SiO_2__0.03	34	103	34.5
Co@SiO_2__0.06	30	97	33.5
Co@SiO_2__0.075	32	100	34.0
Co@SiO_2__0.09	32	97	32.5

The literature also reports synthesized Co@SiO_2_ catalysts with a small total diameter average, between 30 and 40 nm using, the same procedure: a solvothermal method for the core and a modified Stöber method for the shell. These differences can be attributed to variations in heating rates, which influence the nucleation and growth mechanisms.^44^

Figure 2 presents the diffraction patterns of the calcined core–shell. The diffraction peaks observed correspond to the Co_3_O_4_ spinel structure (JCPDS file no. 42–1467), while the diffuse, low-intensity peak around 22° is characteristic of the silica phase. These findings confirm the presence of cobalt oxide (Co_3_O_4_) and silica in the samples. Furthermore, the silica did not exhibit any detectable influence on the crystalline structure of the metallic core particles. A similar behavior was observed for the impregnated material used as a standard for comparison.

**Figure 2:**
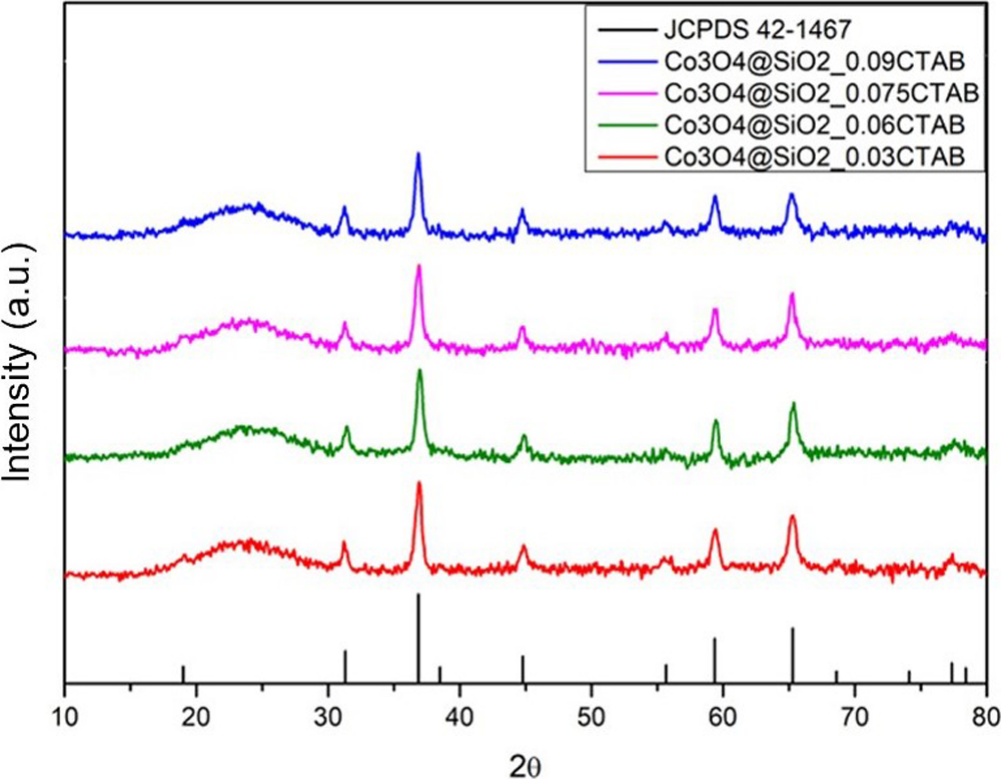
XRD patterns of the calcined core–shell catalysts.

Table 4 presents the average crystallite sizes of Co_3_O_4_ and Co^0^ for all prepared samples, determined by applying the Scherrer equation to the diffraction peak corresponding to the (311) plane. The crystallite size of 14 nm was calculated for the encapsulated catalysts and 15 nm for the supported catalyst. Furthermore, the average crystallite size of metallic cobalt and its dispersion were calculated based on eqs 1 and 2, as detailed in the Experimental Section.

**Table 4:** Crystallite Size of Co_3_O_4_ and Metallic Cobalt

catalysts	2θ (°)	fwhm (°)	$d_{{\mathrm{Co}}_{3}O_{4}}$ (nm)	$d_{{\mathrm{Co}}^{0}}$ (nm)	*D* (%)
Co/SiO2	36.8	0.5	15	11	8.6
Co@SiO_2__0.03	36.9	0.6	14	11	9.2
Co@SiO_2__0.06	36.9	0.6	14	11	9.2
Co@SiO_2__0.075	36.9	0.6	14	11	9.2
Co@SiO_2__0.09	36.9	0.6	14	11	9.2

As observed, the average diameter for the crystallite size of cobalt oxide crystallite was the same for both encapsulated and impregnated samples. This suitable result allows us to analyze the isolated influence of the textural properties on the performance of catalysts. The average crystallite diameter of Co_3_O_4_ obtained by X-ray diffraction is consistent with some reports in the literature when the solvothermal method was used under the same conditions.^33,36,45,46^

To evaluate the reducibility of the materials, the samples Co@SiO_2__0.03 and Co@SiO_2__0.09 were analyzed by temperature-programmed reduction (TPR) under conditions identical to those employed in the reduction step of the catalytic activity tests, that is, in pure H_2_ flow. As illustrated in Figures 3 and 4, the H_2_O formation profiles during TPR analysis reflect the reduction of cobalt oxides in the calcined catalyst material.

**Figure 3:**
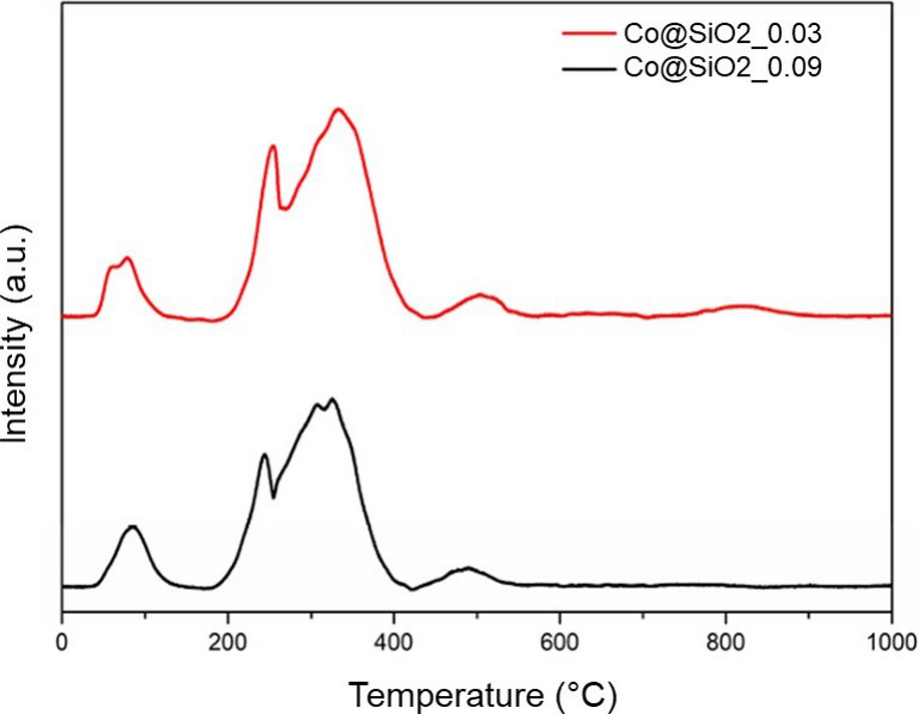
H_2_O formation profiles during the TPR of the Co@SiO_2__0.03 and Co@SiO_2__0.09 catalysts.

**Figure 4:**
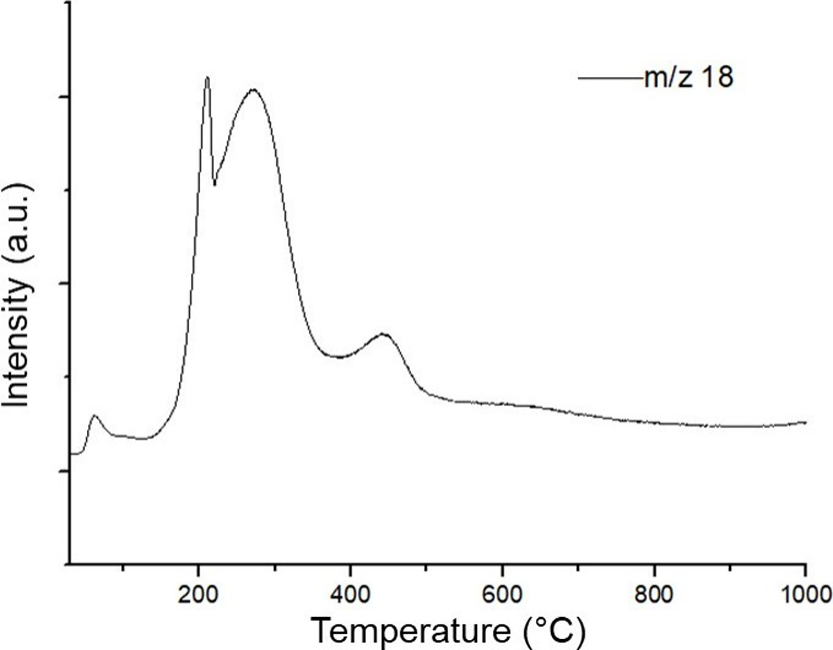
H_2_O formation profiles during the TPR of the supported catalyst.

The reduction profiles shown in Figures 4 and 5 allow us to identify a low-intensity peak, at a temperature close to 100 °C, attributed to possible residual moisture originating from the H_2_ current.^33^ As is well settled, the reduction profile of cobalt oxide involves a two-step reaction, as shown in eqs 6 and 7.

$${\mathrm{Co}}_{3}O_{4}+H_{2}\to 3\mathrm{CoO}+H_{2}O$$ 6

$$\mathrm{CoO}+H_{2}\to {\mathrm{Co}}^{0}+H_{2}O$$ 7

**Figure 5:**
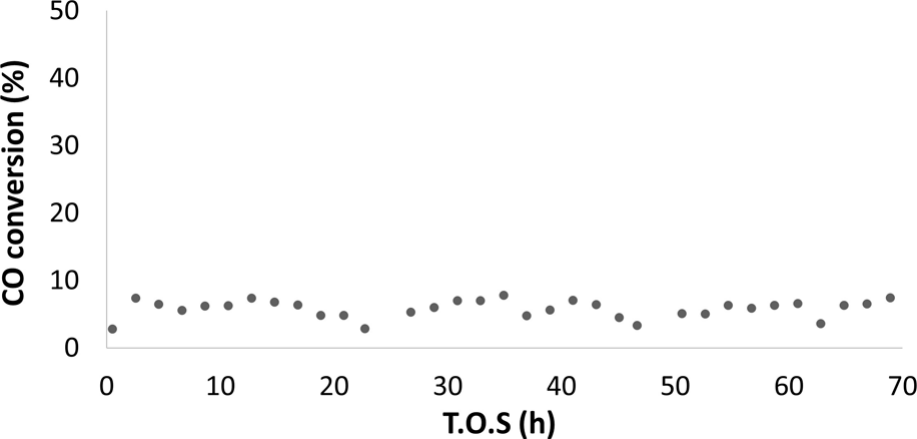
CO conversion with a Co/SiO_2_ catalyst as a function of catalytic test reaction time.

Thus, the two peaks observed at temperatures lower than 400 °C can be attributed to the reduction of Co_3_O_4_ to CoO and from CoO to Co^0^ at temperatures in the range of 200–350 °C. These results are consistent with those obtained in our previous work.^34,47^ Likewise, the peaks observed at higher temperatures, 500 and 830 °C, are associated with the reduction of cobalt silicate species, resulting from the reaction between CoO and the Si–OH groups present in silica.^12,48^

The second peak, associated with the reduction of CoO to Co^0^, shifts to a slightly higher temperature for the encapsulated catalyst when compared to that for the supported catalyst. Literature reports similar shifts, which may indicate a limitation of H_2_ diffusion due to shell encapsulation.^34,38^ However, upon comparison of the two encapsulated catalysts, the Co@SiO_2__0.09 sample did not exhibit a greater limitation to hydrogen diffusion through the silica layer, despite having a considerably smaller average pore diameter. No shift in the reduction peaks to higher temperatures reinforces that statement.

The estimated H_2_ consumption values for Co@SiO_2__0.03, Co@SiO_2__0.09, and Co/SiO_2_ were 1.15, 1.08, and 1.83 mmol/g_cat_, respectively, with corresponding reduction degrees of 80, 87, and 57%. The higher reducibility observed for the encapsulated catalysts suggests a weaker interaction between the metallic oxide nucleus and the silica core–shell. This result indicates that the core–shell morphology reduces the interaction with silica, thereby enhancing the reducibility for metallic oxide crystallites of similar size.

### Fischer–Tropsch Synthesis

The experimental performance tests for the reference catalyst, Co/SiO_2_, revealed a stable conversion of approximately 7% over 72 h of reaction, with low methane selectivity, as shown in Figures 5 and 6. The low degree of reduction of this catalyst, about 57%, likely contributes to its limited value of CO conversion.

**Figure 6:**
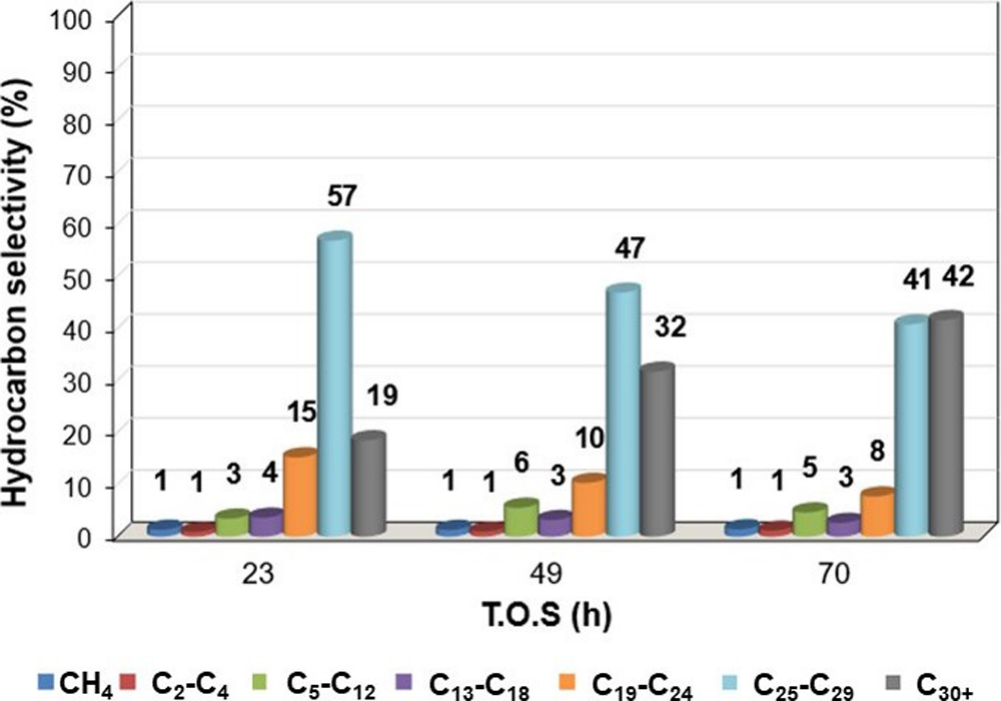
Selectivity after 72 h of Fischer–Tropsch synthesis with a Co/SiO_2_ catalyst.

Regarding product distribution, there was a greater selectivity toward heavier hydrocarbons, specifically in the wax range (C_25_–C_29_ and C_30+_), while maintaining a low methane selectivity (∼1%), which is desirable. Other researchers have reported similar trends. Zhang et al. observed a relatively low CO conversion (∼6.9%), investigating silica-supported cobalt catalysts, attributed to low cobalt dispersion and limited active sites. In contrast, Mu et al. achieved higher CO conversion values (∼ 20%) and significant methane selectivity (∼22%). The authors linked the low reducibility of Co/SiO_2_ to its diminished catalytic activity and increased methane selectivity.

The performance evaluation of the encapsulated samples demonstrated that all were active and stable in the Fischer–Tropsch reaction under the catalytic test conditions. The CO conversion results are listed in Figure 7. For the Co@SiO_2__0.03 sample, an initial CO conversion of approximately 8% was observed, which increased to 15% after 24 h and remained stable at this value until the end of the reaction. The Co@SiO_2__0.06 and Co@SiO_2__0.09 samples exhibited initial CO conversions of approximately 8 and 5%, respectively. After 24 h, their conversion stabilized at around 7 and 4%, respectively, and remained constant throughout the reaction. The Co@SiO_2__0.075 sample was excluded from the tests due to its similarity in pore diameter to the Co@SiO_2__0.06 sample, rendering its inclusion redundant. Figure 8 exhibits the product selectivity after 72 h.

**Figure 7:**
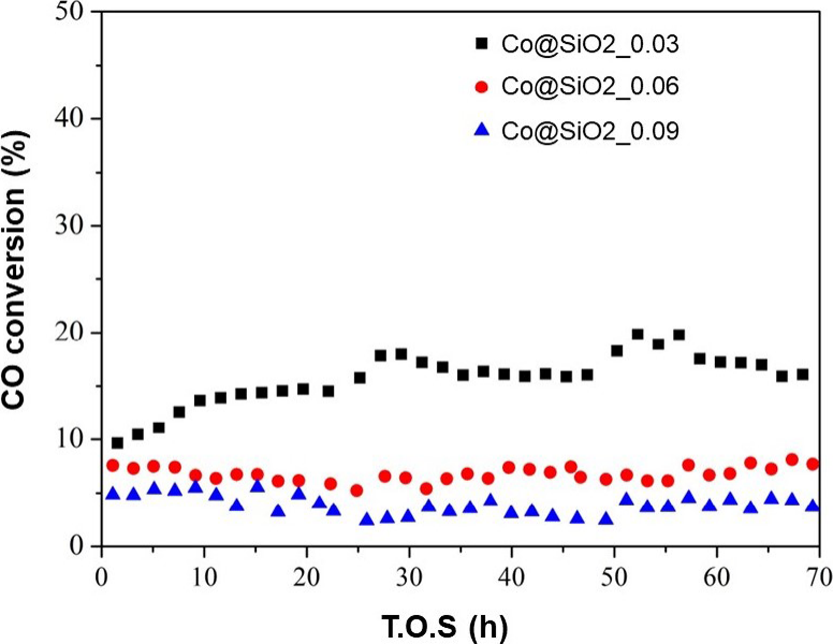
CO conversion with encapsulated catalysts as a function of the catalytic test reaction time.

**Figure 8:**
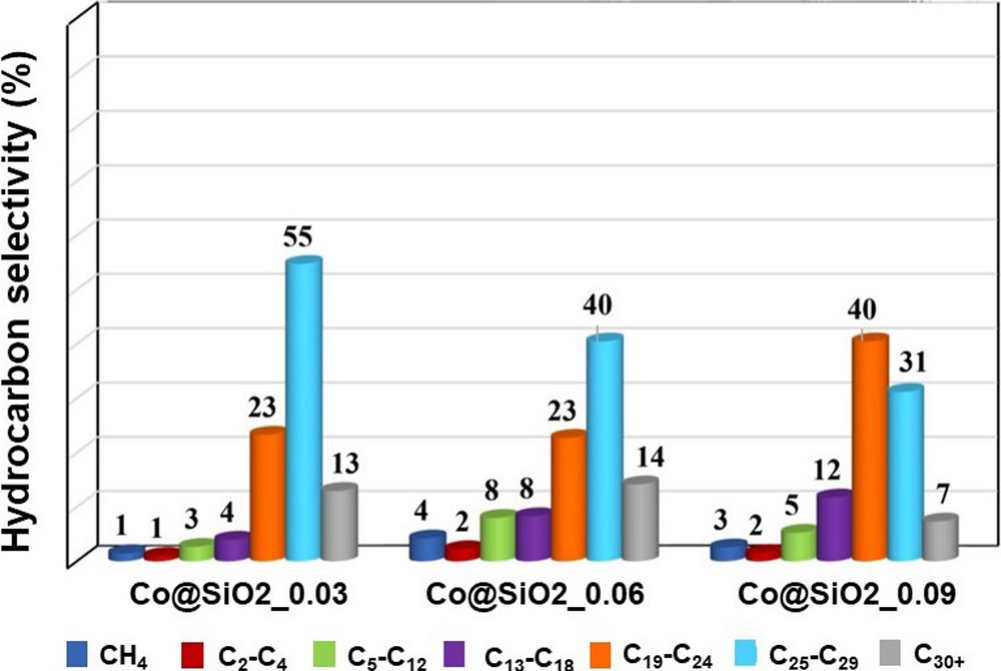
Selectivity after 72 h of Fischer–Tropsch synthesis with encapsulated catalysts.

All of the catalysts investigated exhibited a relatively narrow range of products, preferentially forming long-chain hydrocarbons. This outcome aligns with expectations based on the cobalt particle size and the reaction parameters employed, which favor the production of heavier hydrocarbons while minimizing methane selectivity.^8,49–51^ Moreover, the observed product distribution deviates from the classical Anderson–Schulz–Flory (ASF) model, particularly in the C_1_ and C_10+_ fractions, a behavior that is consistent with deviations previously reported in the literature.^52,53^

These results underscore the potential of FTS to produce a lubricant oil fraction of excellent purity. FTS-derived fuels and lubricants are free of sulfur, nitrogen, and aromatic compounds, offering superior environmental performance. Additionally, this process reduces the carbon footprint compared to conventional oil-derived processes, particularly if utilizing sustainable feedstocks.

The confinement effect is another key aspect to highlight in the core–shell morphology. This effect enhances the contact time between reaction intermediates and active sites, promoting the growth of longer-chain hydrocarbons. Furthermore, the product distribution exhibited significant differences, despite the comparable cobalt particle size and silica shell thickness, particularly within the C_19_–C_24_ and C_25_–C_29_ hydrocarbon fractions. The results revealed that a decrease in the pore diameter correlates with an increase in the C_19_–C_24_ fraction, while the C_25_–C_29_ fraction decreased. When comparing Co@SiO_2__0.03 and Co@SiO_2__0.09, which exhibit extremes in pore diameter (9.2 and 3.5 nm, respectively), the influence of this parameter becomes evident. A reduction in the pore diameter increased the C_19_–C_24_ fraction selectivity by 17%, accompanied by a 24% decrease in the C_25_–C_29_ fraction. This behavior indicates that the observed variations in product distribution are primarily associated with changes in the textural properties of the silica shell, mainly pore diameters.

Likely, the effect of the pore-size diameter on cobalt-supported mesoporous silica has been observed, emphasizing that the average pore size plays a critical role in determining product distribution during hydrocarbon synthesis. Catalysts with an average pore size of approximately 7.5 nm tend to favor the production of long-chain hydrocarbons. In contrast, a smaller pore size, around 3.1 nm, favors short-chain hydrocarbons, possibly because of restricted diffusion and the limited space available for chain growth.^24^

Finally, it should be emphasized that the reaction conversion and selectivity are expected to change when catalysts with distinct morphologies and textural properties are applied. Herein, the supported catalyst demonstrated a selectivity enhancement for heavier hydrocarbons in the C_30+_ range; meanwhile, the encapsulated catalyst, with distinct pore diameter values, gives selectivity for hydrocarbons in the C_19_–C_24_ and C_25_–C_29_ fractions, depending on the pore size. Notably, under similar conditions, all catalysts showed low methane selectivity, highlighting their efficiency in minimizing the formation of undesired light hydrocarbons.

### Spent Catalyst Characterization

Analysis of spent catalysts provides valuable insights into deactivation mechanisms; it allows us to assess their physical and chemical stability, focusing on structural and textural properties and resistance to sintering after the reaction.

STEM images of the spent core–shell catalysts, shown in Figure 9, reveal that the core–shell structure of the encapsulated catalysts was preserved under the applied reaction conditions. This observation confirms the structural stability of the core–shell catalysts.

**Figure 9:**
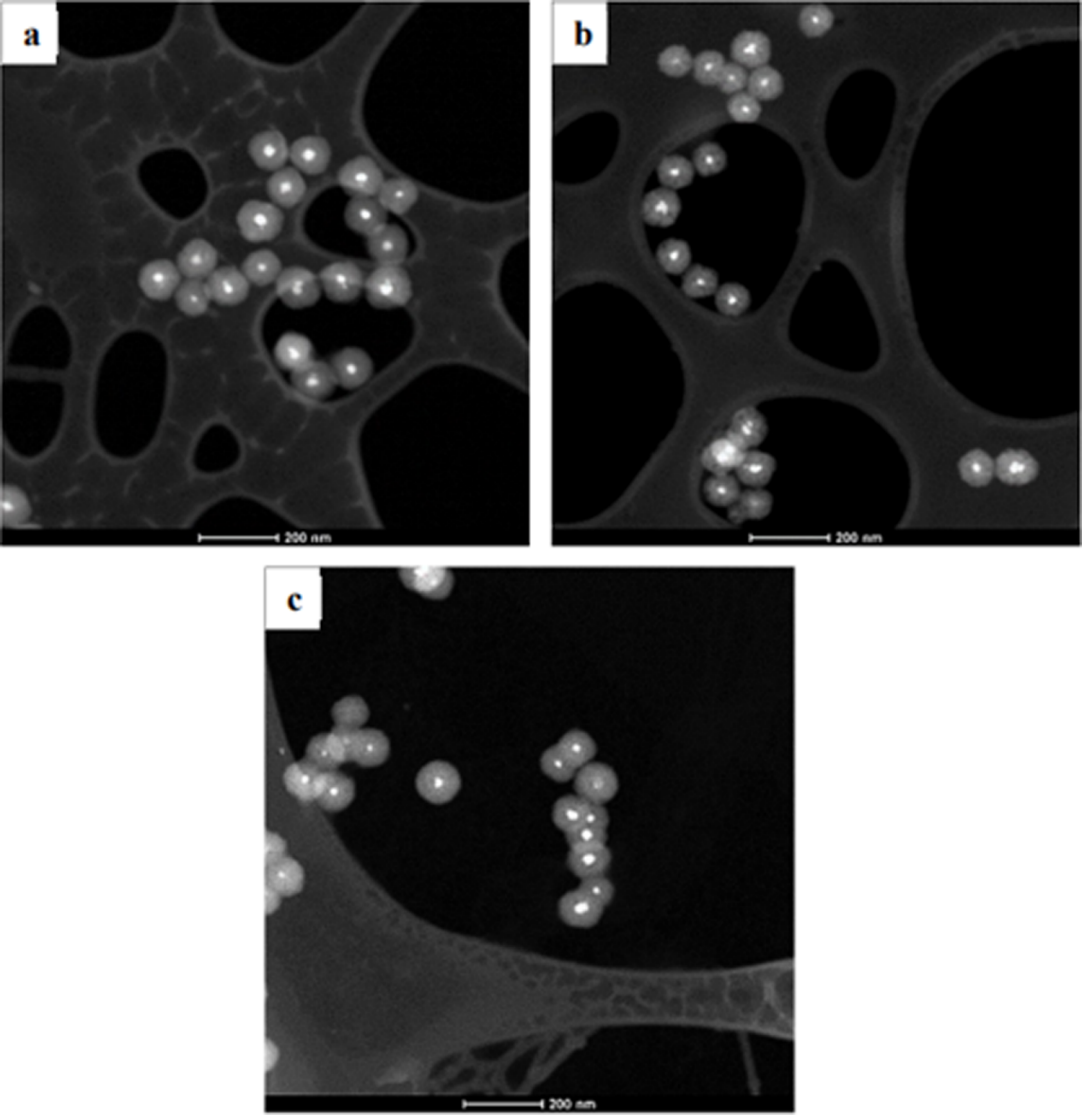
STEM images of the spent (a) Co@SiO_2__0.03, (b) Co@SiO_2__0.06, and (c) Co@SiO_2__0.09 catalysts after Fischer–Tropsch synthesis.

Table 5 presents the average core diameters and the mean whole diameters of the nanoparticles obtained from the spent catalysts. Comparison with the data presented in Table 3, recorded before the reaction, reveals that the total diameter of the particles remained unchanged. The reduction in the average diameter of the nanoparticle metal core aligns with an approximately 25% decrease in crystallite size observed through XRD, attributed to the reduction of Co_3_O_4_ to metallic Co.

**Table 5:** Cobalt Particle Size and Silica Shell Thickness of Spent Catalysts after Fischer–Tropsch Synthesis

samples	core diameter (nm)	total diameter (nm)	thickness of SiO_2_ shell (nm)
Co@SiO_2__0.03	34	103	34.5
Co@SiO_2__0.06	30	97	33.5
Co@SiO_2__0.09	32	97	32.5

These findings are consistent with our previous studies,^34^ reinforcing the sintering resistance of core–shell catalysts and showing the protective effect of the silica shell on the active phase, which mitigates one of the major deactivation players for these catalysts.

## Conclusions

The solvothermal and modified Stöber methods were effective in preparing silica-encapsulated cobalt nanoparticles. Dispersed Co_3_O_4_ nanoparticles were successfully synthesized, with polyvinylpyrrolidone (PVP) as a stabilizing agent, preventing agglomeration during the solvothermal synthesis step. It is worth mentioning that the surfactant content significantly influenced textural properties, affecting pore size, pore volume, and specific surface area. When applied in Fischer–Tropsch synthesis, the core–shell Co@SiO_2_ catalysts, compared to silica-supported cobalt, demonstrated improved performance in the reaction for both selectivity and activity. The Co@SiO_2_ catalyst achieved higher conversion values, particularly with a larger pore diameter, and produced a narrower hydrocarbons distribution, predominantly in the lubricant oil range (C_19_–C_24_ and C_25_–C_29_). Changes in the textural properties of the silica shell had a pronounced effect on the catalytic performance: larger pore diameters enhanced the activity and possibly minimized diffusion limitation. Specifically, the reduction in the pore size from 9.2 nm (Co@SiO_2__0.03) to 3.5 nm (Co@SiO_2__0.09) resulted in a 17% increase in selectivity toward the C_19_–C_24_ fraction and a concomitant 24% decrease in the C_25_–C_29_ fraction. These highlight the critical role of the catalyst’s textural properties in steering the hydrocarbon chain growth, providing a strategic lever for optimizing FT catalysts to favor the production of desired hydrocarbon ranges.

Furthermore, the silica shell provided a protective effect in the active phase, with no evidence of sintering observed in the spent encapsulated catalysts. These results underscore the potential to tailor the synthesis methodology to control the nanostructure by controlling the porosity of the shell. This allows for tuning the selectivity in FTS, as core–shell catalysts act as nanoreactors with porous walls, controlling selectivity.

## Supplementary Material



## Notes

Adsorption and desorption isotherm of the supported catalyst, Co/SiO_2_, adsorption and desorption isotherm of the Co@SiO_2__0.03 catalyst, adsorption and desorption isotherm of the Co@SiO_2__0.06 catalyst, adsorption and desorption isotherm of the Co@SiO_2__0.075 catalyst, and adsorption and desorption isotherm of the Co@SiO_2__0.09 catalyst (PDF)
