# Supplementary material for: Influence of Textural Properties of Co@SiO2 Catalysts on the Performance of Fischer–Tropsch Synthesis
Source: ACS Omega. 2025 Sep 30;10(40):46749–58. doi: 10.1021/acsomega.5c04178 (PMC12529159; doi:10.1021/acsomega.5c04178)
Supplement: Supplementary file 1 [file ao5c04178_si_001.pdf]

Appendix A.
Supplementary Information: Adsorption isotherms of the supported and encapsulated catalysts.

INFLUENCE OF TEXTURAL PROPERTIES OF Co@SiO_2 CATALYSTS ON PERFORMANCE OF FISCHER–TROPSCH SYNTHESIS

Abigail Noemi Esquivel Ojeda,* Camylla M. Moraes, Alexander E. Caytuero-Villegas, Maria Auxiliadora S. Baldanza, Fabio S. Toniolo, Neuman S. de Resende, and Vera M. M. Salim.

Chemical Engineering Program / COPPE, Centro de Tecnologia, Universidade Federal do Rio de Janeiro (UFRJ), Cidade Universitária, Rio de Janeiro, Brazil

E-mail: abigail@peq.coppe.ufrj.br, vera@peq.coppe.ufrj.br

Phone: +55 (21)998742986

For all samples investigated, the N_2 adsorption isotherms were identified as type IV in accordance with the IUPAC classification. Such isotherms exhibit a hysteresis loop associated with capillary condensation, a characteristic feature of mesoporous solids. The hysteresis loop can be classified as type H1, distinguished by two nearly parallel branches of the isotherm and typically attributed to porous materials composed of rigid aggregates of spherical particles with uniform dimensions (Sing et al., 1984). This finding is in good agreement with the morphological features revealed by Scanning transmission electron microscopy (STEM)

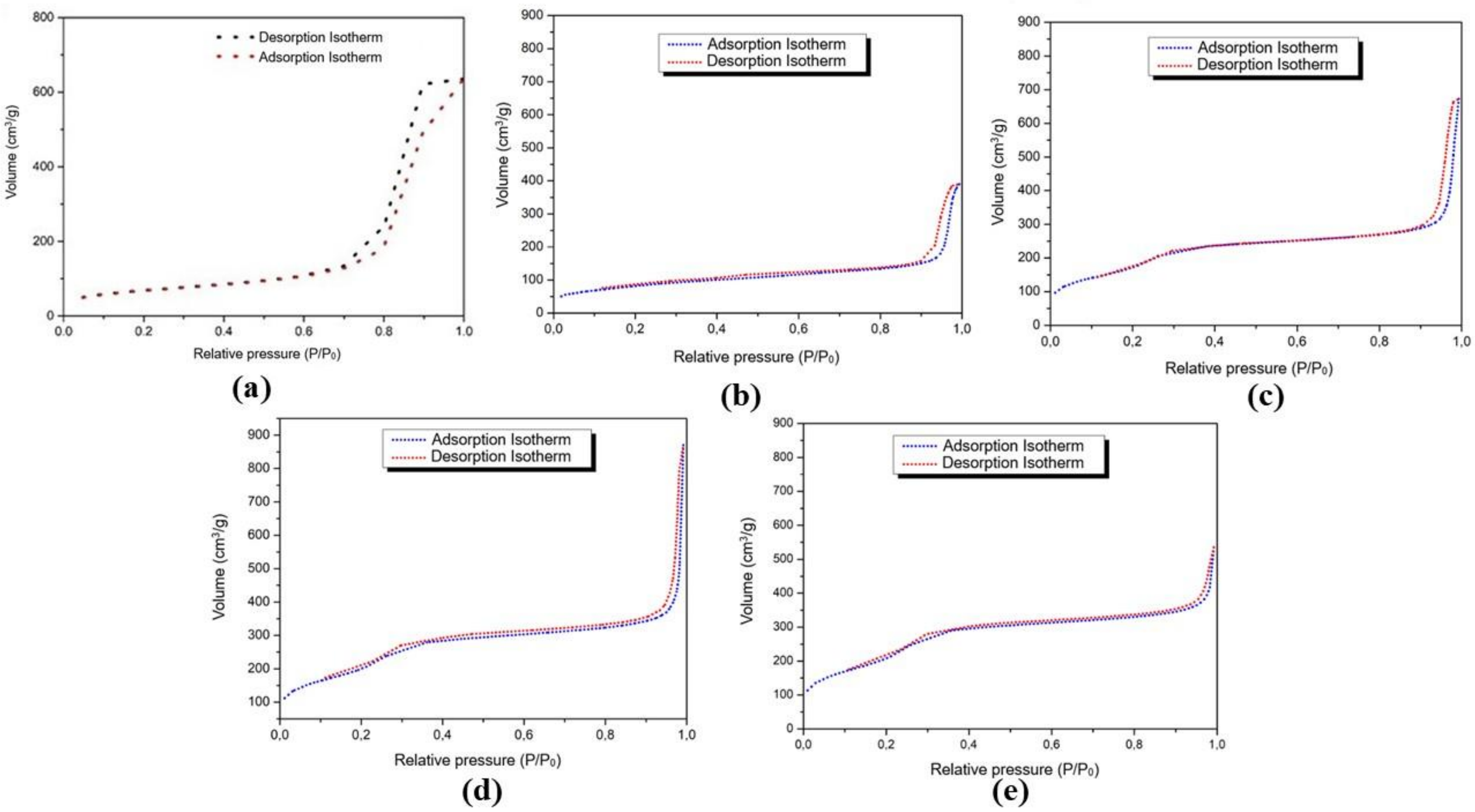

Figure S1: Adsorption and desorption isotherm of the supported catalyst, Co/SiO_2 (a). Adsorption and desorption isotherm of the Co@SiO_2_0.03 catalyst (b). Adsorption and desorption isotherm of the Co@SiO_2_0.06 catalyst (c). Adsorption and desorption isotherm of the Co@SiO_2_0.075 catalyst (d). Adsorption and desorption isotherm of the Co@SiO_2_0.09 catalyst (e).
